# Coronavirus disease 2019 (COVID-19) vaccination rates and staffing shortages among healthcare personnel in nursing homes before, during, and after implementation of mandates for COVID-19 vaccination among 15 US jurisdictions, National Healthcare Safety Network, June 2021–January 2022

**DOI:** 10.1017/ice.2023.87

**Published:** 2023-11

**Authors:** Hannah E. Reses, Minn Soe, Heather Dubendris, George Segovia, Emily Wong, Shanjeeda Shafi, Elizabeth J. Kalayil, Meng Lu, Suparna Bagchi, Jonathan R. Edwards, Andrea L. Benin, Jeneita M. Bell

**Affiliations:** 1 Surveillance Branch, Division of Healthcare Quality Promotion, Centers for Disease Control and Prevention, Atlanta, Georgia; 2 Lantana Consulting Group, East Thetford, Vermont; 3 Goldbelt C6, Chesapeake, Virginia

## Abstract

**Objective:**

To examine temporal changes in coverage with a complete primary series of coronavirus disease 2019 (COVID-19) vaccination and staffing shortages among healthcare personnel (HCP) working in nursing homes in the United States before, during, and after the implementation of jurisdiction-based COVID-19 vaccination mandates for HCP.

**Sample and setting:**

HCP in nursing homes from 15 US jurisdictions.

**Design:**

We analyzed weekly COVID-19 vaccination data reported to the Centers for Disease Control and Prevention’s National Healthcare Safety Network from June 7, 2021, through January 2, 2022. We assessed 3 periods (preintervention, intervention, and postintervention) based on the announcement of vaccination mandates for HCP in 15 jurisdictions. We used interrupted time-series models to estimate the weekly percentage change in vaccination with complete primary series and the odds of reporting a staffing shortage for each period.

**Results:**

Complete primary series vaccination among HCP increased from 66.7% at baseline to 94.3% at the end of the study period and increased at the fastest rate during the intervention period for 12 of 15 jurisdictions. The odds of reporting a staffing shortage were lowest after the intervention.

**Conclusions:**

These findings demonstrate that COVID-19 vaccination mandates may be an effective strategy for improving HCP vaccination coverage in nursing homes without exacerbating staffing shortages. These data suggest that mandates can be considered to improve COVID-19 coverage among HCP in nursing homes to protect both HCP and vulnerable nursing home residents.

Nursing-home residents have been disproportionately affected by the coronavirus disease 2019 (COVID-19) pandemic.^1^ Early in the pandemic, outbreaks of COVID-19 in nursing homes were reported to be caused by asymptomatic and presymptomatic severe acute respiratory syndrome coronavirus 2 (SARS-CoV-2) infections in residents and healthcare personnel (HCP).^2,3^ High rates of SARS-COV-2 infection among HCP have also strained the healthcare workforce and exacerbated staffing shortages.^4^

In May 2020, the Centers for Medicare & Medicaid Services (CMS) released interim final rules to ensure routine reporting of COVID-19 cases among residents and HCP in CMS-certified nursing homes.^5,6^ In May 2021, CMS issued final rules for COVID-19 vaccine reporting requirements for residents and HCP. By July and August 2021, 15 US jurisdictions enacted mandates requiring nursing home staff to be vaccinated against COVID-19 as a condition of employment.^7^

The impact of jurisdiction-based mandates for vaccination against COVID-19 on HCP vaccine coverage and staffing shortages warrants assessment to inform future decisions about mandates for vaccination. Due to differences across jurisdictions in enforcement, religious and medical exemptions, and offers of a SARS-CoV-2 testing alternative in lieu of vaccination, it was not evident whether mandates would increase primary series vaccination against COVID-19 among HCP.^8^ In addition, before the announcement of jurisdiction-based mandates, HCP working in jurisdictions that enacted a mandate had higher baseline vaccination against COVID-19 compared to HCP working in jurisdictions that did not enact a mandate (71.6% vs 56.1%, respectively),^9^ generating uncertainty around whether the mandates, or other factors, led to increases in coverage in these jurisdictions. Concerns were raised about the possibility of vaccine mandates exacerbating existing challenges with staffing shortages.^10–12^ This assertion appeared to be supported by repeated news reports of HCP leaving their jobs and protesting vaccine mandates that were adopted by private and public organizations across the country.^12–14^

In this study, we examined the temporal change in completed primary series vaccination against COVID-19 and staffing shortages among HCP working in nursing homes before, during, and after the implementation of 15 jurisdiction-based mandates using data from the Centers for Disease Control and Prevention’s (CDC) National Healthcare Safety Network (NHSN) from June 7, 2021, through January 2, 2022.

## Methods

### Data source

The CDC NHSN began national, facility-level surveillance for COVID-19 cases and staffing shortages in nursing homes in May 2020 and COVID-19 vaccination reporting in December 2020. The NHSN is a national surveillance program for healthcare-associated infections, vaccinations of healthcare facility staff and nursing home residents, and other patient safety metrics.^9^ COVID-19 surveillance data reported by nursing homes to the NHSN include cases of COVID-19 among residents and HCP, testing practices, resident occupancy, staffing shortages, and vaccination coverage among residents and HCP. These data are reported weekly and are due 1 week after the end of a reporting week.

Complete primary series COVID-19 vaccination was defined as receiving dose 1 and dose 2 of COVID-19 vaccines requiring 2 doses for completion or 1 dose of COVID-19 vaccine requiring only 1 dose for completion, at this facility or elsewhere since December 2020.^15^ Staffing shortages were defined according to the facility’s determination of what constitutes a shortage of HCP.^9^

Individuals with a medical contraindication were excluded from the denominator for the calculation of the percentage of HCP with complete primary series vaccination. A medical contraindication to COVID-19 vaccination was defined as history of a severe allergic reaction (eg, anaphylaxis) after a previous dose or to a component of the COVID-19 vaccine, or history of a known diagnosed allergy to a component of the COVID-19 vaccine.^9^ HCP were defined as those who were eligible to have worked at this healthcare facility for at least 1 day during the week of data collection, regardless of clinical responsibility or patient contact.^9^

Information on jurisdiction-based COVID-19 vaccine mandates for HCP were obtained from a Kaiser Family Foundation dashboard and webpage summarizing US jurisdiction COVID-19 policy actions (Table 1).^7^ From this site, we abstracted information on jurisdiction-based mandate status for nursing home HCP (yes vs no) and a description of the mandate, including whether the jurisdiction provided a test-out option for HCP to be routinely tested for COVID-19 in lieu of receiving vaccination. Among the 15 jurisdictions that announced mandatory vaccination policies for HCP working in nursing homes, we searched the Internet to obtain the policy announcement date and mandate deadline for complete primary series vaccination, or we approximated it if only the first dose deadline had been announced.

**Table 1.** Healthcare Personnel Vaccination Mandate Periods for Complete Primary Vaccine Series by Jurisdiction—National Healthcare Safety Network, June 7, 2021–January 2, 2022

Jurisdiction	Nursing Homes	Announcement Date	Mandate Deadline	Mandate Period, Days^a^
California	1,206	7/27/2021	10/31/2021	82
Colorado	225	8/10/2021	10/29/2021	78
Connecticut^b^	209	8/6/2021	10/18/2021	70
District of Columbia^b^	17	8/16/2021	10/18/2021	60
Delaware	45	8/12/2021	10/10/2021	67
Illinois	709	8/4/2021	10/1/2021	52
Massachusetts	375	8/4/2021	10/27/2021	72
Maryland^2^	230	8/18/2021	10/1/2021	44
Maine	93	8/12/2021	10/7/2021	62
New Jersey	364	8/2/2021	10/7/2021	51
New Mexico	69	8/17/2021	9/7/2021	36
New York^2^	623	8/16/2021	10/5/2021	62
Oregon	130	8/19/2021	9/30/2021	65
Rhode Island	80	8/10/2021	9/30/2021	49
Washington	201	8/9/2021	10/30/2021	75
Median	209	8/10/2021	10/7/2021	62
Range	17–1,206	7/27/21–8/19/21	9/7/21–10/31/21	36–82

a No. of days from announcement to deadline.

b Four jurisdictions only announced a deadline for healthcare personnel (HCP) to receive the first dose of a COVID-19 primary series. For these jurisdictions, we estimated the approximate deadline by adding 4 weeks to the first-dose deadlines announced.

### Sample and setting

In this study, we included nursing homes from 15 US jurisdictions (California, Colorado, Connecticut, District of Columbia, Delaware, Illinois, Massachusetts, Maryland, Maine, New Jersey, New Mexico, New York, Oregon, Rhode Island, Washington) that announced and enacted HCP vaccination mandates during July and August 2021. We restricted selection to CMS-certified nursing homes that reported to the NHSN weekly COVID-19 vaccination modules in the 15 jurisdictions with mandates from June 7, 2021, through January 2, 2022. The study period was selected to provide at least 4 weeks of observation time before and after the announcement of each mandate. The week of June 7–13, 2021, was considered the baseline week before any jurisdictions announced a vaccination mandate for HCP.

### Statistical analysis

To determine the temporal impact of jurisdiction-based mandates on change in primary series vaccine coverage and the odds of reporting a staffing shortage, we divided the study period into three time periods: (1) preintervention period (before the week of the announcement of the mandate); (2) intervention period (from the week of the mandate announcement through the week of the mandate deadline); and (3) postintervention period (after the week of the mandate deadline for complete primary series vaccination). The announcement and deadline dates varied by jurisdiction. Therefore, the periods were examined separately for each jurisdiction.

We analyzed data at the facility level assessing 2 primary outcome metrics: (1) HCP with complete primary series vaccination divided by the number of all HCP working in the facility (excluding those with a medical contraindication) each week; and (2) the odds of a nursing home reporting a staffing shortage each week. We constructed interrupted time-series (segmented regression) models^16^ to obtain estimates overall (all 15 jurisdictions combined) and for each jurisdiction. We incorporated a generalized estimating equation (GEE) method^17^ with an autoregressive (AR1) correlation matrix structure to account for serial correlation (autocorrelation) errors from repeated measures of primary series vaccination coverage and staffing shortages reported from the same facility and obtained robust standard errors of the regression coefficients. For each week, we calculated a rate ratio of the current week vaccination coverage divided by the prior week vaccination coverage. We used negative-binomial log-linear models to estimate weekly percent change of vaccination coverage [(vaccination coverage rate ratio − 1) × 100 per 1-week increase in time] for each period.^16^ We assessed interaction of slopes between the preintervention and intervention periods and between the intervention and postintervention periods to assess whether the weekly percentage change in complete primary series vaccination differed by period with respect to the mandate announcement and deadline.

We used logistic regression models to estimate an odds ratio (the odds of a facility reporting a staffing shortage per one week increase in time) for each period. We then assessed interaction of slopes between the preintervention and intervention periods and between the intervention and postintervention periods to estimate the temporal change in the odds of a nursing home reporting a staffing shortage.

We also assessed bed capacity of the nursing homes and community-level factors, including the CDC Social Vulnerability Index,^18^ the NCHS urban–rural classification scheme,^19^ and county-level community COVID-19 incidence, as potential confounders in all models. However, these factors were excluded from the final models because they did not alter the parameter estimates of the outcome metrics by >10%. We performed model fit and diagnostics by examining fit statistics and residual graphs to assess high leverage, outliers, and influential data points. We conducted all analyses using SAS version 9.4 software (SAS Institute, Cary, NC), and we defined statistical significance at α = .05 (2 tailed). This activity was reviewed by the CDC and was conducted consistently with applicable federal law and CDC policy.

## Results

In total, 4,576 nursing homes (96.7%) reported HCP vaccination data from the 15 jurisdictions with a COVID-19 vaccination mandate for HCP during the study period. We obtained complete primary series vaccination deadlines for 11 of the 15 jurisdictions and estimated approximate deadlines for the remaining 4 jurisdictions by adding 4 weeks to the first-dose deadline. Among the 15 jurisdictions, announcement and deadline dates for HCP vaccination varied, with a median announcement date of August 8, 2021, and a median deadline of October 7, 2021 (Table 1). The mandate period between announcement and deadline dates ranged from 36 to 82 days, with a median of 62 days.

### Staff vaccination

Overall, primary series vaccine coverage among HCP increased over time from 66.69% at baseline (week ending June 13, 2021) to 94.28% at the end of the study period (week ending January 2, 2022). The combined regression model showed the vaccination rate among HCP increased by 0.67% per week (95% confidence interval [CI], 0.59–0.75) before the intervention period and significantly accelerated to 2.17% per week (95% CI, 2.11–2.24) during the intervention period, and subsequently increased by 0.68% per week (95% CI, 0.65–0.72) in the postintervention period (Table 2). The interaction terms between the preintervention and intervention periods and between the intervention and postintervention periods were statistically significant (*P* < .0001), indicating the weekly rate of change differed across the 3 periods.

**Table 2.** Temporal Change in Complete Primary Series Vaccination Coverage Rate Among Nursing Home Healthcare Personnel Before, During, and After Jurisdiction-Based Vaccination Mandate, Based on Overall (All Jurisdictions Combined) and Jurisdiction-Specific Regression Models—National Healthcare Safety Network, June 7, 2021–January 2, 2022

Jurisdiction	No. of Nursing Homes	Study Period	Weekly % Change	95% CI (Lower Limit)	95% CI (Upper Limit)	*P* Value	Interaction Term *P* Value
Combined	4,576	Preintervention period	0.67	0.59	0.75	<.0001	
		Intervention period	2.17	2.11	2.24	<.0001	<.0001^a^
		Postintervention period	0.68	0.65	0.72	<.0001	<.0001^b^
California	1,206	Preintervention period	0.71	0.54	0.88	<.0001	
		Intervention period	1.49	1.37	1.61	<.0001	<.0001^a^
		Postintervention period	0.33	0.29	0.38	<.0001	<.0001^b^
Colorado	225	Preintervention period	0.95	0.69	1.20	<.0001	
		Intervention period	2.29	2.07	2.52	<.0001	<.0001^a^
		Postintervention period	0.45	0.33	0.58	<.0001	<.0001^b^
Connecticut	209	Preintervention period	1.01	0.79	1.23	<.0001	
		Intervention period	2.90	2.61	3.19	<.0001	<.0001^a^
		Postintervention period	0.37	0.27	0.48	<.0001	<.0001^b^
District of Columbia	17	Preintervention period	2.07	1.02	3.13	.0001	
		Intervention period	1.10	0.36	1.84	.0033	.0405^a^
		Postintervention period	0.36	0.06	0.67	.0192	.0459^b^
Delaware	45	Preintervention period	1.75	1.16	2.34	<.0001	
		Intervention period	1.14	0.14	2.15	.0257	.2866^a^
		Postintervention period	1.40	0.96	1.85	<.0001	.5317^b^
Illinois	709	Preintervention period	1.28	1.06	1.51	<.0001	
		Intervention period	1.77	1.58	1.95	<.0001	.0005^a^
		Postintervention period	1.30	1.18	1.42	<.0001	<.0001^b^
Massachusetts	375	Preintervention period	0.99	0.80	1.18	<.0001	
		Intervention period	2.29	2.11	2.48	<.0001	<.0001^a^
		Postintervention period	0.41	0.31	0.50	<.0001	<.0001^b^
Maryland	230	Preintervention period	1.06	0.79	1.34	<.0001	
		Intervention period	1.65	1.27	2.03	<.0001	.0137^a^
		Postintervention period	0.66	0.51	0.81	<.0001	<.0001^b^
Maine	93	Preintervention period	0.61	0.34	0.88	<.0001	
		Intervention period	2.58	2.24	2.93	<.0001	<.0001^a^
		Postintervention period	0.62	0.39	0.85	<.0001	<.0001^b^
New Jerseu	364	Preintervention period	1.32	1.08	1.56	<.0001	
		Intervention period	1.27	1.00	1.54	<.0001	.6944^a^
		Postintervention period	1.06	0.95	1.16	<.0001	.2963^b^
New Mexico	69	Preintervention period	1.22	0.78	1.67	<.0001	
		Intervention period	3.62	3.07	4.18	<.0001	<.0001^a^
		Postintervention period	0.20	0.00	0.40	.0489	<.0001^b^
New York	623	Preintervention period	1.20	1.06	1.33	<.0001	
		Intervention period	3.10	2.96	3.23	<.0001	<.0001^a^
		Postintervention period	0.65	0.59	0.71	<.0001	<.0001^b^
Oregon	130	Preintervention period	0.33	0.11	0.55	.0035	
		Intervention period	2.36	2.02	2.71	<.0001	<.0001^a^
		Postintervention period	0.54	0.36	0.72	<.0001	<.0001^b^
Rhode Island	80	Preintervention period	0.51	0.13	0.90	.009	
		Intervention period	2.79	2.06	3.52	<.0001	<.0001^a^
		Postintervention period	0.81	0.51	1.11	<.0001	<.0001^b^
Washington	201	Preintervention period	0.98	0.63	1.32	<.0001	
		Intervention period	2.50	2.20	2.79	<.0001	<.0001^a^
		Postintervention period	0.37	0.26	0.48	<.0001	<.0001^b^

a Interaction term *P* value for preintervention and intervention study periods.

b Interaction term *P* value for intervention and postintervention study periods.

**Fig. 1.**
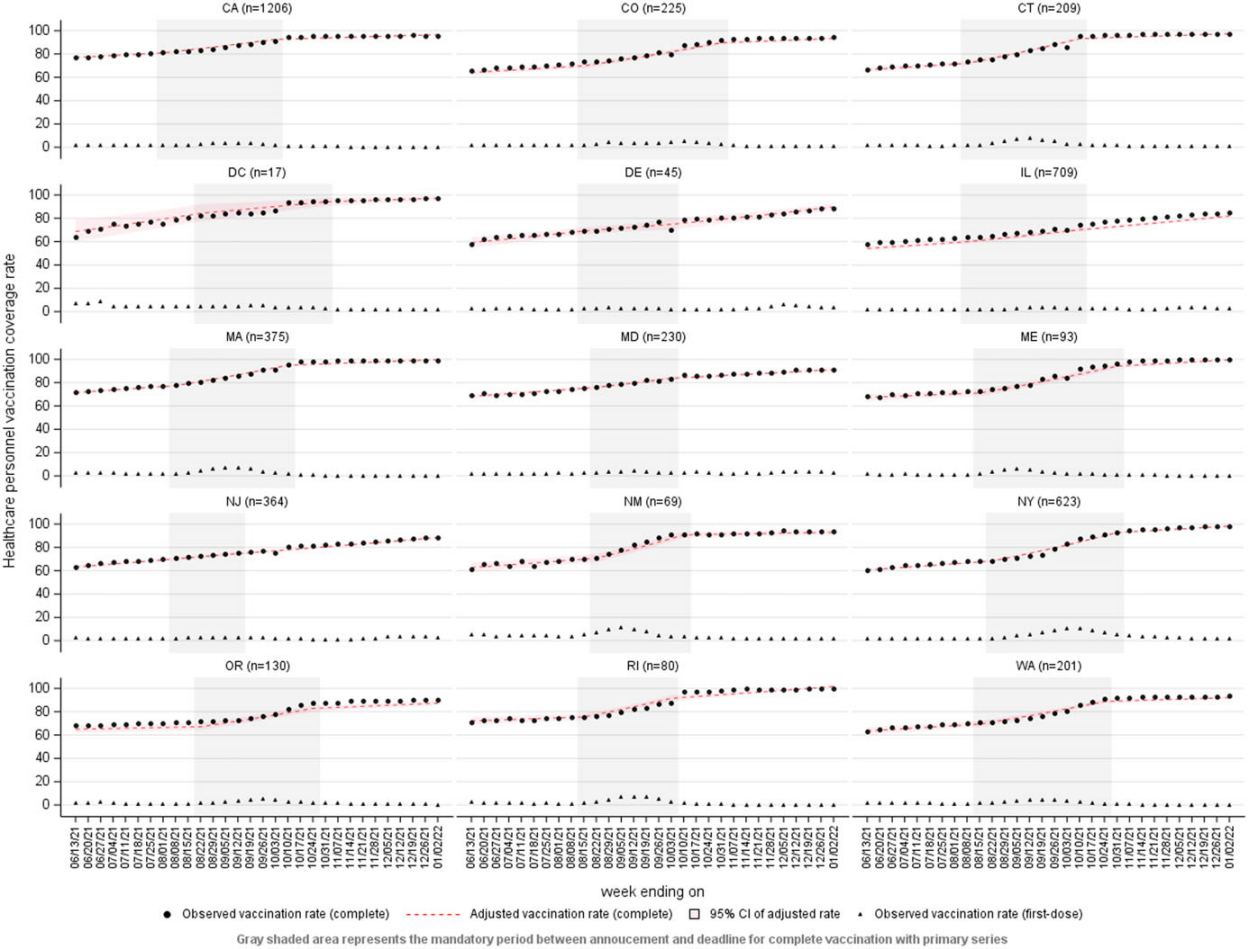
Interrupted time-series plots of pooled mean coverage with a complete primary series of COVID-19 vaccination among nursing home healthcare personnel (HCP) by jurisdiction, National Healthcare Safety Network, June 7, 2021–January 2, 2022. The gray shaded area in each plot represents the intervention period between the mandate announcement and mandate deadline dates. Note: Black circles, observed weekly complete primary series vaccination coverage; red dashes, adjusted weekly complete primary series vaccination coverage; transparent red shading, 95% confidence interval of adjusted weekly complete primary series vaccination coverage; black triangles, observed weekly partial primary series vaccination coverage (first dose of a two-dose primary series).

The jurisdiction-specific models showed similar trends, with complete primary series vaccine coverage increasing in individual jurisdictions from 57.90%–77.44% at baseline to 84.97%–99.75% at the end of the study period (Table 2, Fig. 1). In 12 of 15 jurisdiction-specific models, the rate of increase in primary series vaccine coverage was highest during the intervention period. In addition, there was statistically significant interaction between period and the weekly rate of change in primary series vaccine coverage in most jurisdictions, indicating that the weekly rate of change differed across periods and was significantly higher during the intervention period than the preintervention period. The first-dose vaccination rate among HCP also significantly increased in most jurisdictions during the first 4 weeks of the intervention period (*P* < .05) (Fig. 1).

However, 3 jurisdictions did not follow this trend. In New Jersey and Delaware, interaction between period and weekly change in vaccine coverage was not detected. In the District of Columbia, there was significant interaction between period and change in vaccine coverage, but the weekly rate of increase was highest in the preannouncement period.

### Staffing shortages

In the combined staffing shortage model, the odds of a nursing home reporting a staffing shortage were highest before the announcement of mandates. The odds increased slightly per week before the mandate announcement (odds ratio [OR], 1.09; 95% confidence interval [CI], 1.08–1.1), but the change in odds per week was low during the intervention period (OR, 1.02; 95% CI, 1.02–1.03) and null after the mandate deadline (OR, 0.99; 95% CI, 0.99–1.00) (Table 3). We detected a statistically significant interaction between period and the weekly change in odds of reporting a staffing shortage, indicating that the weekly change in odds differed across the periods, with the weekly increase in odds being highest in the preannouncement period and lower during and after the mandate period.

**Table 3.** Temporal Change in the Odds of Nursing Homes Reporting Staff Shortage Before, During, and After Jurisdiction-Based Vaccination Mandate, Based on Overall (All Jurisdictions Combined) and Jurisdiction-Specific Regression Models—National Healthcare Safety Network, June 7, 2021–January 2, 2022

Jurisdiction	No. of Nursing Homes	Study Period	Odds Ratio	95% CI (Lower Limit)	95% CI (Upper Limit)	*P* Value	Interaction Term *P* Value
Combined	4,576	Preintervention period	1.09	1.08	1.10	<.0001	
		Intervention period	1.02	1.02	1.03	<.0001	<.0001^a^
		Postintervention period	0.99	0.99	1.00	.0057	<.0001^b^
California	1,206	Preintervention period	1.02	0.98	1.07	.2588	
		Intervention period	1.02	0.99	1.05	.1912	.9336^a^
		Postintervention period	1.00	0.98	1.02	.7362	.4193^b^
Colorado	225	Preintervention period	1.07	1.04	1.11	<.0001	
		Intervention period	1.01	0.99	1.03	.2696	.0044^a^
		Postintervention period	1.02	1.00	1.04	.123	.7612^b^
Connecticut	209	Preintervention period	1.06	1.00	1.13	.0444	
		Intervention period	0.98	0.93	1.03	.377	.0633^a^
		Postintervention period	1.03	0.98	1.08	.3013	.2705^b^
District of Columbia	17	Preintervention period	1.05	0.96	1.14	.2637	
		Intervention period	1.02	0.91	1.13	.7817	.7024^a^
		Postintervention period	1.08	0.94	1.24	.2956	.6114^b^
Delaware	45	Preintervention period	0.96	0.91	1.01	.1087	
		Intervention period	1.00	0.90	1.12	.9677	.5387^a^
		Postintervention period	1.07	1.02	1.13	.0109	.3807^b^
Illinois	709	Preintervention period	1.02	1.00	1.04	.0989	
		Intervention period	1.02	1.01	1.04	.0048	.7627^a^
		Postintervention period	1.01	1.00	1.02	.079	.3506^b^
Massachusetts	375	Preintervention period	1.06	1.00	1.13	.0626	
		Intervention period	1.01	0.96	1.06	.6676	.2671^a^
		Postintervention period	1.06	1.03	1.09	.0004	.2074^b^
Maryland	230	Preintervention period	1.01	0.99	1.04	.2402	
		Intervention period	0.98	0.95	1.01	.226	.148^a^
		Postintervention period	1.02	1.00	1.05	.021	.0548^b^
Maine	93	Preintervention period	1.03	1.01	1.06	.0076	
		Intervention period	1.04	1.02	1.07	.0018	.644^a^
		Postintervention period	1.01	0.98	1.04	.4359	.1315^b^
New Jersey	364	Preintervention period	1.05	0.99	1.11	.0747	
		Intervention period	1.03	0.98	1.09	.223	.6902^a^
		Postintervention period	1.04	1.01	1.06	.0014	.9415^b^
New Mexico	69	Preintervention period	1.01	0.97	1.05	.598	
		Intervention period	1.06	1.00	1.12	.0699	.2617^a^
		Postintervention period	0.99	0.96	1.02	.4141	.0663^b^
New York	623	Preintervention period	1.04	1.02	1.06	<.0001	
		Intervention period	1.04	1.03	1.06	<.0001	.944^a^
		Postintervention period	1.03	1.02	1.05	<.0001	.411^b^
Oregon	130	Preintervention period	1.04	1.00	1.07	.0257	
		Intervention period	0.99	0.96	1.01	.333	.0494^a^
		Postintervention period	1.02	0.99	1.04	.2292	.2068^b^
Rhode Island	80	Preintervention period	1.00	0.97	1.04	.8361	
		Intervention period	1.04	0.99	1.10	.1271	.3198^a^
		Postintervention period	1.02	0.98	1.05	.3228	.4591^b^
Washington	201	Preintervention period	1.04	1.02	1.07	.0008	
		Intervention period	1.02	0.99	1.04	.174	.1443^a^
		Postintervention period	1.02	1.00	1.04	.0389	.6895^b^

a Interaction term *P* value for preintervention and intervention study periods.

b Interaction term *P* value for intervention and postintervention study periods.

**Fig. 2.**
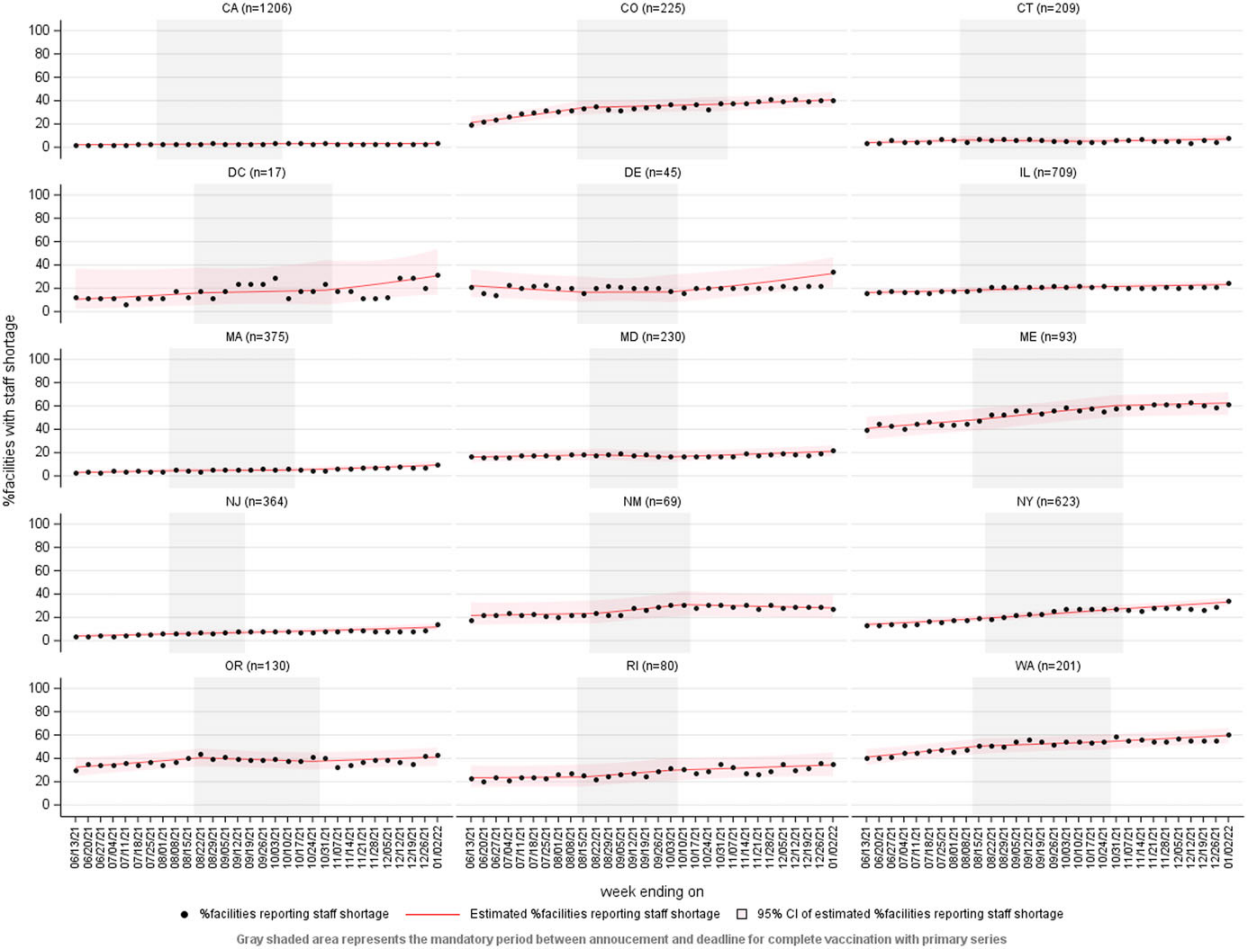
Interrupted time series plots of percentage of nursing homes reporting a staffing shortage by jurisdiction, National Healthcare Safety Network, June 7, 2021– anuary 2, 2022. The gray shaded area in each plot represents the intervention period between the mandate announcement and mandate deadline dates. Note: Black circles, observed weekly percentage of nursing homes reporting a staffing shortage; red lines, adjusted weekly percentage of nursing homes reporting a staffing shortage; transparent red shading, 95% confidence interval of adjusted weekly percentage of nursing homes reporting a staffing shortage.

In the jurisdiction-specific models, the weekly change in the odds of a nursing home reporting a staffing shortage did not differ across the 3 periods (*P* > .05 for interaction terms between the preannouncement and mandate periods and between the mandate and postdeadline periods), apart from Colorado and Oregon. In these jurisdictions, interaction between period and the weekly change in the odds of a nursing home reporting a staffing shortage was detected, with the highest weekly increase in odds in the preannouncement period (Table 3 and Fig. 2).

## Discussion

In this study, we focused on temporal impacts of jurisdiction-based mandates for vaccination for HCP in nursing homes in the United States before, during, and after mandate implementation. In jurisdictions that mandated primary series vaccination against COVID-19 for HCP working in nursing homes, primary series vaccination increased at the fastest rate during the period between the announcement of the mandate and the deadline, suggesting a meaningful response to the mandates. Moreover, the weekly change in the odds of a nursing home reporting a staffing shortage were lowest after the mandate period, suggesting jurisdiction-based requirements for HCP COVID-19 vaccinations may be effective for improving vaccination rates in nursing home HCP without introducing staffing shortages. Recent studies similarly found that jurisdiction-level mandates for HCP vaccination were associated with higher vaccination coverage in HCP and were not associated with increases in reported staffing shortages.^8,20^ Our finding that staffing shortages were lowest after the intervention period is consistent with other study findings that increases in COVID-19 vaccination among HCP may improve staffing conditions by significantly reducing HCP morbidity, SARS-CoV-2 infection, absenteeism, and duration of absenteeism during periods of high SARS-CoV-2 circulation in the community.^21^

Vaccine mandates for HCP are not a novel strategy for reducing morbidity and mortality associated with vaccine-preventable diseases in healthcare settings. Many US healthcare facilities require that HCP receive other vaccines, such as the influenza vaccine, varicella vaccine, and the measles, mumps, and rubella vaccine.^22^ Similar to our findings, employer mandates for other vaccinations have been reported to be associated with higher coverage among HCP.^22–27^ For example, in the 2019–2020 influenza season, an estimated 81% of HCP in the United States received influenza vaccine, with higher coverage among HCP who were required by their employer to be vaccinated (94%) than those whose employer did not require vaccination (70%).^28,29^

Although our data show that the rate of increase in vaccine coverage was highest during the mandate period in 12 of 15 jurisdictions, 3 jurisdictions (District of Columbia, Delaware, and New Jersey) did not experience an increase in vaccine uptake after the mandates were announced. Notably, policies in Delaware and New Jersey provided a test-out option, meaning that HCP who wanted to remain unvaccinated for any reason could undergo additional COVID-19 testing in lieu of receiving primary series vaccination against COVID-19. These results are consistent with those from a study in which increases in vaccination coverage were largest when jurisdiction-level mandates had no test-out option, suggesting that strict mandates are most effective.^8^ Test-out options may diminish the effect of vaccination mandates in increasing vaccine coverage rates. Compared to receiving vaccination, test-out is also inferior in reducing exposure to HCP and residents because of limitations associated with this strategy (eg, who is testing [self vs employer], timeliness of testing, and poor point of care test sensitivity leading to false negatives). Moreover, the risk of morbidity and mortality is higher among unvaccinated individuals compared to vaccinated individuals, which may independently exacerbate staffing shortages.^30^

International studies conducted to assess the impact of COVID-19 vaccination mandates on HCP vaccination coverage have also shown complementary results. A federal COVID-19 vaccine mandate in Italy was implemented on April 8, 2021, for all HCP.^31^ As of August 2021, 94.4% of HCP received both primary COVID-19 vaccine doses, compared to 50.9% of the general population aged 12–79 years.^32^ This difference is suggestive of the impact of COVID-19 vaccine mandates on increasing vaccination among HCP because vaccination requirements for the general population in Italy (eg, employment mandates, public/private business mandates) did not commence until September 2021.^33^ An additional study in an Italian hospital demonstrated a decrease in the number of sick days used by HCP after receiving complete COVID-19 primary series vaccination compared to prevaccination, suggesting that vaccine mandates may strengthen healthcare workforce capacity rather than exacerbate shortages.^34^ This finding is consistent with our finding that staffing shortages were lowest in the postmandate period.

Our study had several limitations. First, collection of data was not primarily designed to evaluate a causal relationship between jurisdiction policies and HCP vaccination coverage or staffing shortages. We were not able to mitigate possible residual confounding effects due to other unmeasured factors such as differences in policies and enforcement by individual facilities and jurisdictions. Second, study outcomes were jurisdiction-level aggregates of pooled mean HCP vaccination coverage rates and staffing shortages; thus, they did not reflect facility-level impacts. Finally, staffing shortage results do not account for whether facilities had to hire temporary workers to prevent shortages they otherwise would have experienced due to the mandate.

We plan to continue assessing the impact of COVID-19 vaccine mandates, including the federal mandate on staff vaccination in the nursing home and in other healthcare facilities including hospitals.^35^ Future analyses may also consider the impact of mandates on HCP vaccination rates including booster dose coverage and other outcomes such as COVID-19 case incidence. Our findings suggest the effectiveness of mandates in improving HCP vaccination coverage without worsening, and potentially improving, staffing shortage levels in nursing homes. Jurisdictions could consider additional measures in nursing homes such as mandates for updated bivalent doses to strengthen workforce capacity and protect both residents and HCP given the continuous evolution of new variants of SARS-CoV-2 virus and waning immunity from prior vaccines and infections.^36^ This may be especially important in the context of the end of the federal COVID-19 public health emergency.^37^
